# Patterns of livestock depredation and Human–wildlife conflict in Misgar valley of Hunza, Pakistan

**DOI:** 10.1038/s41598-021-02205-2

**Published:** 2021-12-07

**Authors:** Rubina Bano, Akbar Khan, Tahir Mehmood, Saeed Abbas, Muhammad Zafar Khan, Arshad Ali Shedayi, Sher Zaman, Muhammad Ali Nawaz

**Affiliations:** 1 Department of Biological Sciences, Karakoram International University, Gilgit, Gilgit-Baltistan, Pakistan; 2 School of Natural Sciences (SNS), National University of Sciences and Technology (NUST), Islamabad, Pakistan; 3 Environmental Sciences, Karakoram International University, Gilgit, Gilgit-Baltistan, Pakistan; 4 Department of Physics, Karakoram International University, Gilgit, Gilgit-Baltistan, Pakistan; 5 Department of Biological and Environmental Sciences, Qatar University, Doha, Qatar

**Keywords:** Ecology, Zoology

## Abstract

Throughout the world, livestock predation by mammalian carnivores causes significant economic losses to poor farmers, and leads to human–wildlife conflicts. These conflicts result in a negative attitude towards carnivore conservation and often trigger retaliatory killing. In northern Pakistan, we investigated livestock depredation by large carnivores between 2014 and 2019, and subsequent Human–wildlife conflict, through questionnaire-based surveys (n = 100 households). We used a semi-structured questionnaire to collect data on livestock population, depredation patterns, predation count, and conservation approaches. We found a statistically significant increasing pattern of predation with influential factors such as age, gender, occupation, education of respondents, population of predators, threats index for predators and conservation efforts. Some 310 livestock heads with an average of 51 animals per year out of the total 9273 heads were killed by predators, and among them 168 (54%) were attributed to the wolf and 142 (45.8%) to snow leopard. Major threats to carnivores in the area included retaliatory killing, habitat destruction and climate change. Incentivization against depredation losses, guarded grazing and construction of predator-proof corral may reduce Human–wildlife conflict and both livelihood and predator can be safeguarded in the study area.

## Introduction

Human–wildlife conflict relates to negative interactions between people and wildlife that can result in injury or death to humans, loss of economic potential and assets, and can culminate in retaliatory killing of the wildlife itself. Predation of livestock is an important factor affecting successful coexistence of large carnivores and humans from pastoral communities^1–4^. Predation on livestock is the primary source of Human–wildlife conflict and considered one of the biggest challenges for conservation of predators that overlap with shared grazing lands^5–7^. In the Karakoram-Pamir mountains, predation has emerged as a serious issue with spatial variation in its intensity, depending upon wild and domestic prey abundance, prey ages, herding practices, and species of predator^8^. As populations of domestic livestock increase, there is an apparent increase in predation and the subsequent retaliatory killing of large carnivores by pastoralists^8–10^. The carnivore population around the globe is declining due to various reasons including conflict with humans, habitat loss, transmission of diseases and loss of natural prey^11,12^ due to illegal hunting and poaching.

Large carnivores in the Karakoram region include the iconic snow leopard (*Uncia uncia*), Tibetan wolf (*Canis lupes*), and Himalayan lynx (*Lynx lynx*)^13^. Other carnivores like the Brown bear (*Ursus arctos*), Tibetan red fox (*Vulpes vulpes vulpes*), Stone martin (*Martes foina*) are also present, but are rarely predators of livestock^8^. The snow leopard is a threatened species in the highland ecosystem of Central Asia^14,15^. The depredation pattern of livestock by large carnivores varies from place to place, given the type and densities of livestock, wild prey-base, spatial and temporal aspects of pastures and herding practices^16^. In the Karakoram, livestock constitute a primary item in the diet of both snow leopards (66.6%) and wolves (75.1%)^17^. The economic loss due to depredation in Hindu Kush, Karakoram and Himalaya region is quite significant like in upper Mustang region of Nepal, it accounts for US$ 44,213 per year^7^. Similarly, US$ 12,252 per year is lost in Jigme Singye Wangchuck National Park^18^ of Bhutan and loss of US$ 12,905 per year has been recorded for Hushey valley in Central Karakorum National Park, Pakistan^19^.

Though there are records of frequent predation cases in the remote areas of Karakoram but still there is lack of appropriate documented data about the scale and scope of Human–wildlife conflict and its consequences. Being one of the buffer zone valley of Khunjerab National Park and a potential wildlife corridor between Pakistan, China and Tajikistan, Misgar hosts a variety of predators which make the valley vulnerable to livestock depredation. The average household income of communities residing in Misgar is less than US$ 500 per year and in a such a scenario, loss of one or two livestock heads per family means a lot for the affected herding family and that in most of the cases ends in retaliatory killing. Therefore, in the current study, we investigated the scale and scope of Human–wildlife conflict in Misgar valley so that we could be able to inform decision makers about site specific conservation strategies.

## Material and methods

### Study area

Misgar is in the northwestern region of district Hunza, Gilgit-Baltistan, Pakistan, adjoining to the Khunjerab National Park (KNP) towards the north-east. Misgar Valley lies at 36° 46′ 34′′ N and 74° 45′ 56′′ E, at 8000 ft. above sea level. The population of the valley is 3000 people and it shares a border with both China and Afganistan^20^. The vast meadows of the valley provide grazing grounds to a large herd of livestock and wild ungulates, and offer important habitat for predators such as wolves, snow leopards, and red fox.

### Data collection

**Figure 1:**
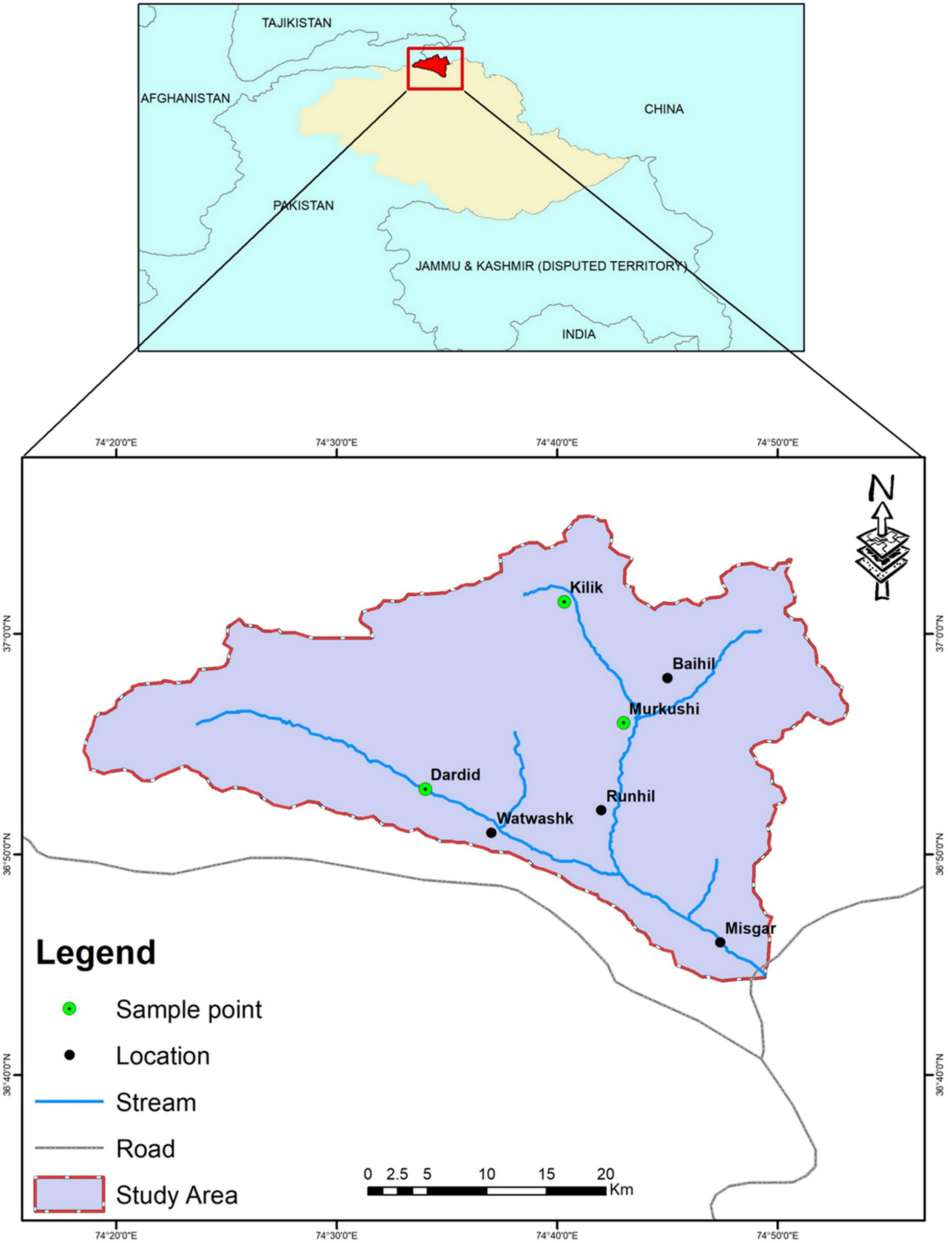
Map of study area, showing locations of livestock depredation. Map is developed in ArcGIS Version 10.8 (www.esri.com).

For data collection, questionnaires were designed for household level (HH) data and HH level interviews and focus group discussion (FGDs) were conducted as earlier used by various researchers^19,21^. Out of 270 households, 100 (37%) households from the predation hotspots like Kilike, Murkushi and Dardee areas of Misgar Valley (Fig. 1) were selected randomly for data collection on scale and scope of livestock predation between 2014 and 2019. The interviews were conducted following an informed consent of the respondents and overall the study plan was approved by the graduate research management committee of the Karakoram International University. In addition to this, all methods were performed in accordance with the relevant guidelines and regulations.

### Data analysis

Data collected on different parameters of Human–wildlife conflict was analyzed in R software (R Development Core Team, 2021), and poisson regression^22,23^ was run to evaluate effects of socio-ecological covariates on livestock predation:

$$log\left(Predation\, Counts\right)={\beta }_{0}+\sum {\beta }_{i}\left(Demeographic \,factor\right)+\sum {\beta }_{j}\left(seasonal \,factor\right)+\sum {\beta }_{k}\left(government \,policiy \,factor\right)+\sum {\beta }_{k}\left(ecosystem\, factor\right)+\sum {\beta }_{k}\left(economic\, factor\right)+\epsilon .$$

Here ${\beta }^{^{\prime}}$ s is the regression coefficient and $\epsilon$ is the model residual. Models were run separately for the snow leopard and wolf predation counts using the poisson regression model. We considered more than 22 different factors that have direct or indirect impact on predation dynamics, which included demographics, seasonal information, government policies, ecosystem, education, occupation, economic situation etc. Snow leopard and wolf predation counts were separately modelled through poisson regression. Moreover, the stepwise model section algorithm helped in designing the parsimonious model, significantly identified the influential factors and showed the best statistical performance.

## Results

### Patterns of livestock depredation

**Figure 2:**
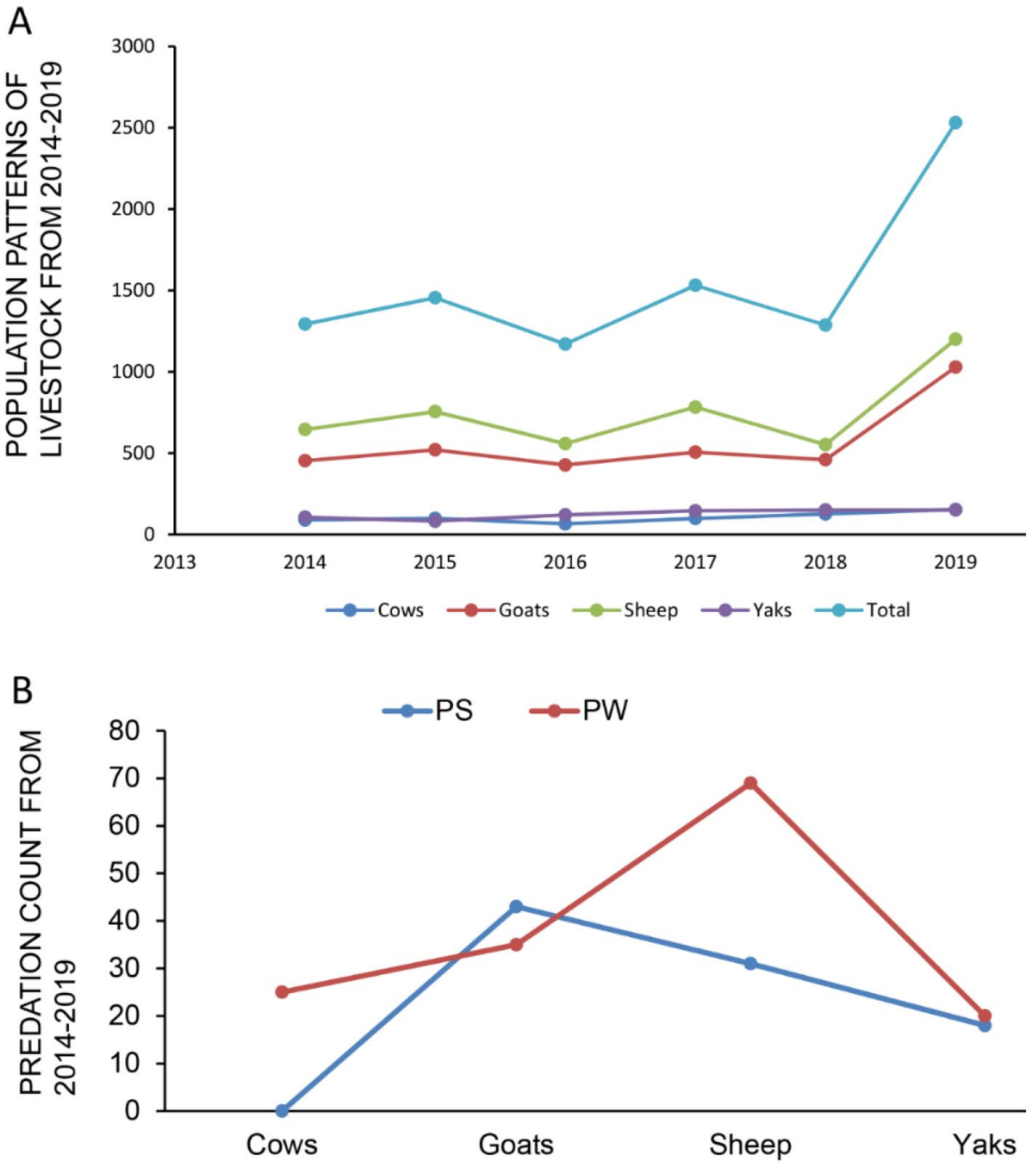
livestock population pattern and predation count. (**A**) Shows 5 years’ population patterns of livestock from 2014 to 2019. (**B**) Shows the predation count by snow leopard (PS), Predation by wolf (PW).

A total of 310 out of 9273 livestock heads were killed by predators in the predation hotspots of Kilike, Murkushi, and Dardee, and among them 142 (45.80%) attributed to snow leopard and 168 (54%) were attributed to the wolf (Table 1, Fig. 2A,B) between 2014 and 2019. Out of the total kills, sheep constitutes highest with (35.16%), followed by goat cattle (27.74%)**,** domestic yak (20.64%) and cattle (16.45%) (Table 2).

**Table 1:** Economic cost of predation by snow leopard and wolf in Misgar valley, district Hunza, during years 2014–2019.

Livestock type	Total livestock toll by predators	Predation by SL	Predation by Wolf	Unit price (PKR)	Estimated loss in PKR	Total loss in US$
Cattle	51	24	27	45,000	2,295,000	14,343.75
Goats	86	47	39	20,000	1,720,000	10,750
Sheep	109	38	71	15,000	1,635,000	10,218.75
Yaks	64	33	31	80,000	5,120,000	32,000
Total	310	142 (45.8%)	168 (54%)	160,000	10,770,000	67,311.75

Average estimated price for predated livestock established village conservation scheme, 1US$ = 160.

*PKR* Pakistan Rupee, *SL* snow leopard.

**Table 2:** Livestock holding in Misgar valley, district Hunza, during years 2014–2019.

Years	Types of livestock owned by respondents				Total	% increase = 100 × final-initial/initial
	Number of cattle	Number of goat	Number of sheep	Number of yak		
2014	89	453	645	106	1293	–
2015	98	520	755	82	1455	–
2016	65	427	558	120	1170	–
2017	98	506	783	145	1532	–
2018	126	459	552	150	1287	–
2019	153	1229	1200	154	2536	37.64%*
Total	629 (6.78%)	3394 (36.6%)	4493 (48.45)	757 (8.16)	9273	–
Predation	51 (16.45)	86 (27.74)	109 (35.16%)	64 (20.64%)	310 (3.34%)	–

*Percent increase after 4 years’ period.

**Figure 3:**
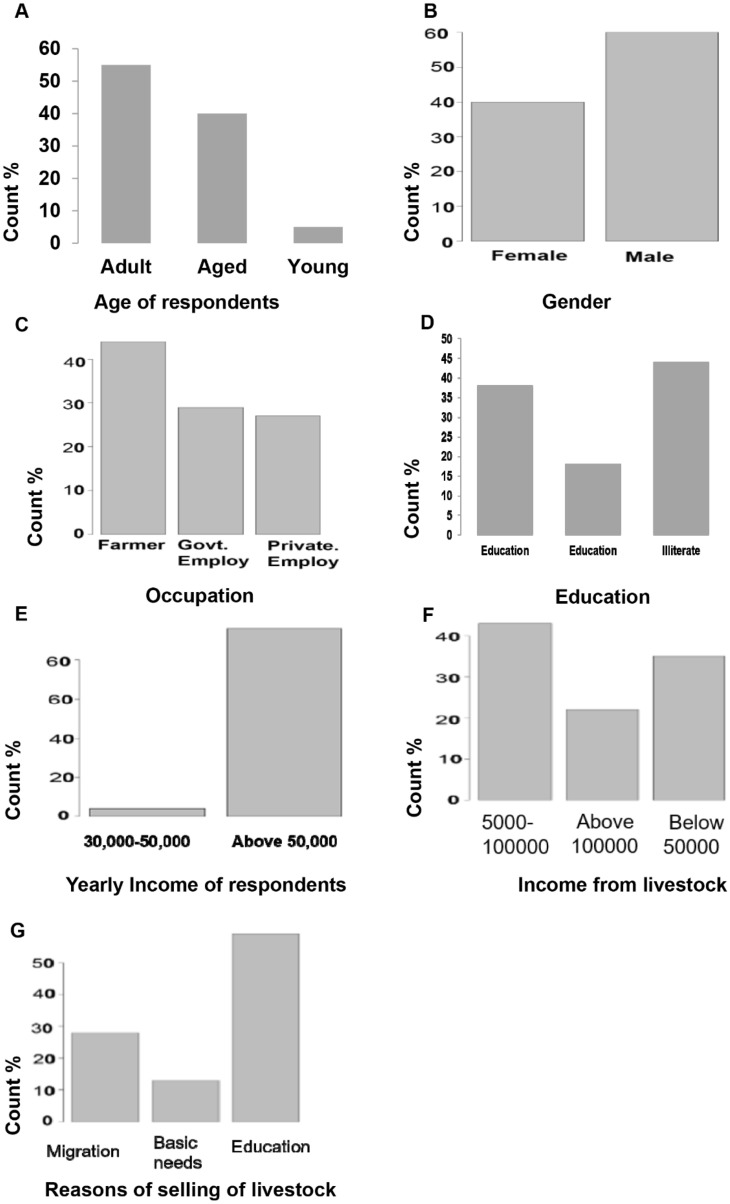
Socioecological profile of the of community residing at Misger Valley.

**Figure 4:**
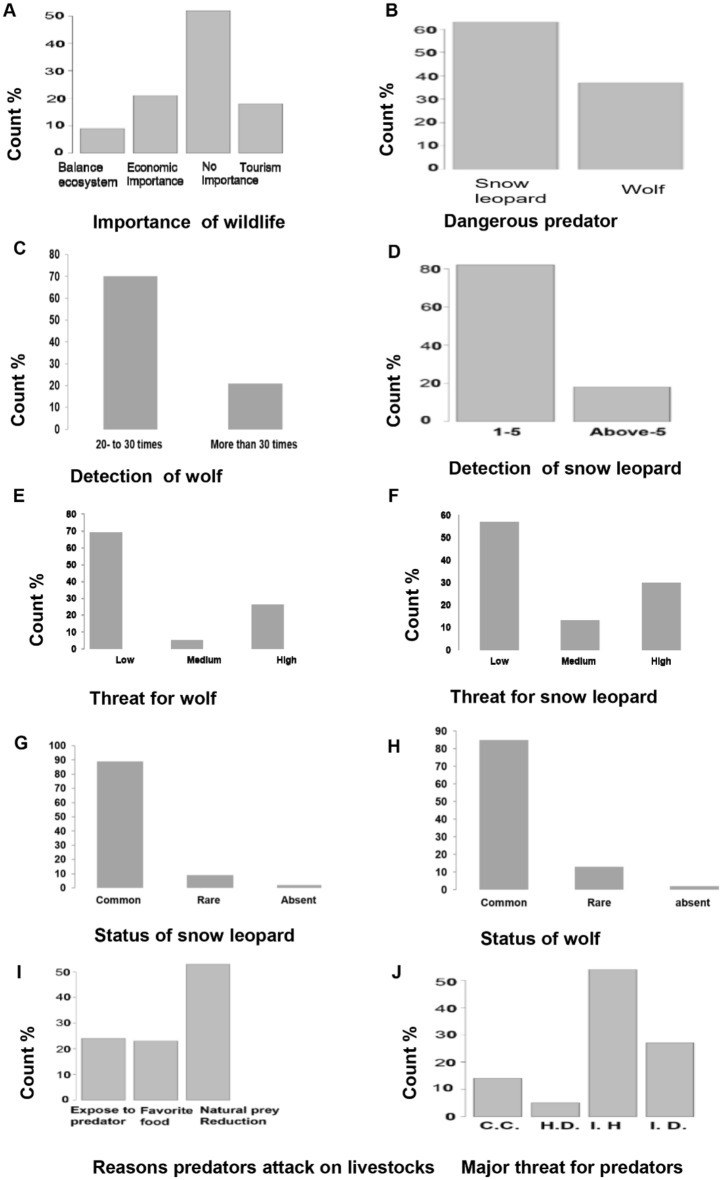
Circumstances of predation and perception of local community towards predators at Misger Valley. Major threats to wildlife (J) were categorized into climate change (CC), habitat destruction (HD), illegal hunting (IH), illegal hunting and habitat degradation (ID).

**Figure 5:**
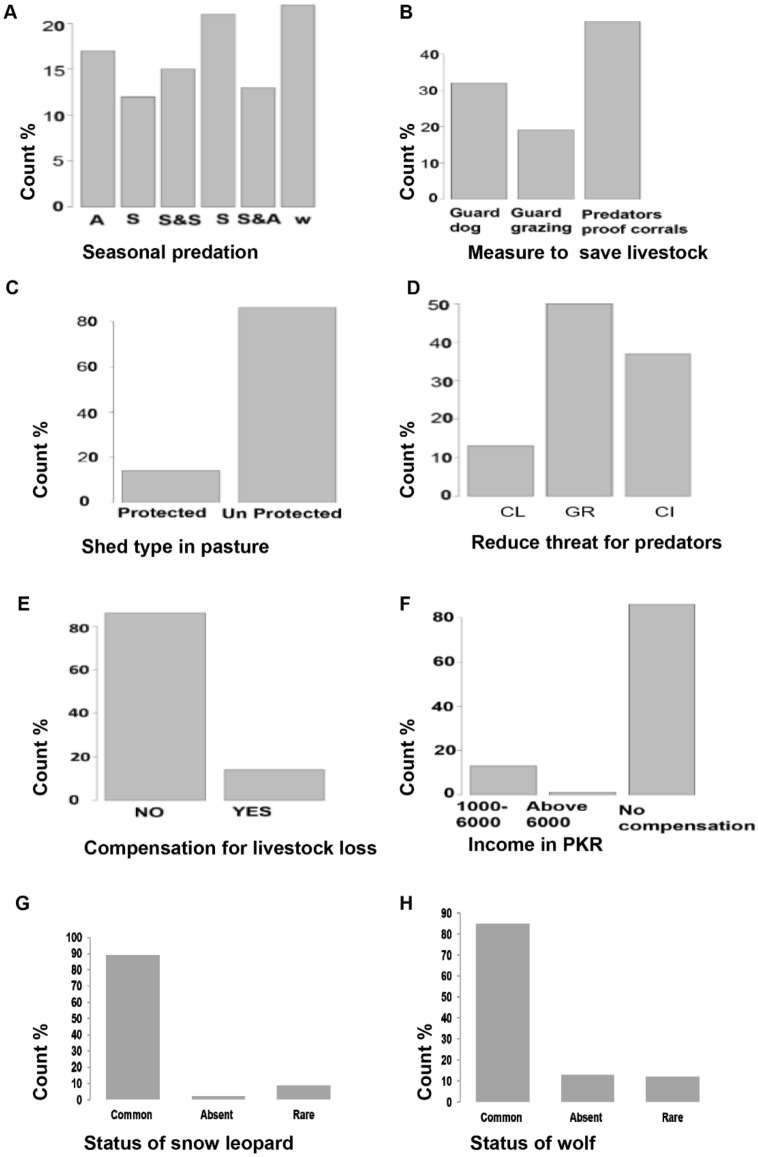
Conservation strategies towards the protection of predators in Misger Valley. Seasonal predation (**A**) is categorized as autumn (A), summer (S), spring and summer (S&S), spring (S), summer and autumn (S&A). Effects of protection measures for livestock and pastures are presented in **B** and **C**, respectively. Mitigation measures (**D**) included conservation laws (CL), Government rules (GR), community involvement (CI). Graphs **E**–**H** show compensation for losses, household income, population status of snow leopard and wolf, respectively.

Results indicates age of respondent, occupation of respondent, gender of respondent, education of respondent, annual income of respondent, estimated income from livestock, reasons of selling live stoke (Fig. 3A–G), protective measures, number of wolf, reasons of attack on livestock, wildlife threats (Fig. 4A–H) and season, measure to save livestock, compensation for livestock lost, income earned as compensation, wolf and snow leopard status appeared influential factors in explaining the variation in snow leopard wolf a predation (Fig. 5A–H). Reference to adult age of respondent, the wolf predation is 0.0795 times less likely for aged respondents (p-value 0.002) and the wolf predation is 3.5 times more likely for young respondent (p-value < 0.01). Reference to basic education, the wolf predation is 0.032 times less likely for respondents having higher education (p-value < 0.001) and the wolf predation is 0.011 times less likely for illiterates (p-value < 0.001). Reference to farmers the wolf predation is 0.056 times less likely for government employees (p-value < 0.001) and is 0.034 times less likely for private employee (p-value < 0.001). The wolf predation decreases for male respondents by 0.039 times (p-value < 0.001). The wolf predation decreases for high income respondents by 0.001 times (p-value < 0.001). Reference to guarded dog the wolf predation is 6.23 times more likely with guarded grazing and is 0.43 times less likely with roof corrals (p-value < 0.001). The wolf predation decreases with high number of wolfs by 0.017 times (p-value = 0.016). The wolf predation decreases with laws of wild life by 0.003 times (p-value < 0.001). Reference to laws for conservation the wolf predation decreases by 0.036 times with government rules (p-value < 0.001). Reference to exposed to predators the wolf predation increases by 2.34 times with favorite food (p-value < 0.001). Reference to autumn the wolf predation decreases by 0.006 times in winter (p-value < 0.001). Reference to the middle income the wolf predation decreases by 0.04 times with the high income (p-value < 0.004). Reference to absent wolf status the wolf predation decreases by 0.193 times with common wolf status (p-value < 0.001) (Tables 3, 4).

**Table 3:** Snow leopard predation in relation to various socio-ecological factors in Misgar valley, district Hunza.

Factors	Levels	Odds ratio	p-value	Factors	Levels	Odd ratio	p-value
**Snow leopard predation**							
Age of respondents	Adult	Reference		Threat for snow leopard	High	Reference	
	Aged	1.03	0.869		Low	1.179	0.346
					Medium	0.578	
	Young	0.16	< 0.001	Major threats for predators	Climate change	Reference	0.076
					Habitat destruction	0.773	0.585
Occupation of owner	Farmer	Reference			Illegal hunting	0.391	< 0.001
	Government employee	6.081	< 0.001		Illegal hunting and habitat destruction	0.185	< 0.001
	Private employee	7.173	< 0.001	Reasons of attack on livestock	Exposed to predator	Reference	
Gender of respondents	Female	Reference			Favorite food	3.207	< 0.001
	Male	0.652	0.042		Natural prey reduction	1.038	0.862
Yearly income	Below 50,000	Reference		Season	Autumn	Reference	
					Spring	1.425	0.302
	Above 50,000	0.055	< 0.001		Summer	0.759	0.305
					Winter	1.799	0.023
Number of snow leopard	1–5	Reference		Protective measures	Guarded dogs	Reference	
	Above 5	0.43	< 0.001		Guarded grazing	1.638	0.051
Snow leopard status	Absent	Reference			Predator proof corrals	0.549	0.002
	Common	2.788	0.005	Condition shed in pasture	Protected	Reference	
	Rare	1.74 2	0.07		Unprotected	0.466	0.003

**Table 4:** Wolf predation in relation to various socio-ecological factors in Misgar valley, district Hunza.

Factors	Levels	Odd ratio	p-value	Factors	Levels	Odd ratio	p-value
**Wolf predation**							
Education of respondent	Basic education	Reference		Laws for wildlife	No	Reference	
	Higher education	0.032	< 0.001		Yes	0.003	< 0.001
	Illiterate	0.011	< 0.001	Reduce wildlife threat	Laws for conservation	Reference	
Age of respondents	Adult	Reference			Gov. rules	0.036	< 0.001
	Aged	0.795	0.002		Local community	0.162	0.774
	Young	3.5	< 0.001	Reasons of attack on livestock	Exposed to predators	Reference	
Occupation of respondent	Farmer	Reference			Favorite food	2.34	< 0.001
	Government employee	0.056	< 0.001		Natural prey reduction	0.689	0.149
	Private employee	0.034	< 0.001	Season	Autumn	Reference	
Gender of respondent	Female	Reference			Spring	0.068	0.826
	Male	0.039	< 0.001		Summer	0.878	0.145
Yearly income of respondent	Below 50,000	Reference			Winter	0.006	< 0.001
	Above 50,000	0.001	< 0.001	Estimate income from livestock	50.000—100,000	Reference	
Protective measures	Guarded dog	Reference			Above 100,000	0.005	0.004
	Guarding grazing	6.23	< 0.001		Below 50,000	0.028	0.064
	Predator proof corrals	0.43	< 0.001	Wolf status	Absent	Reference	
Number of wolf	1–30	Reference			Common	0.193	< 0.001
	Above 30	0.017	0.016		Rare	0.31	0.541

About 54% of the respondents claimed that illegal hunting is the major threat followed by illegal hunting and habitat destruction with 32% and climate change is threatening about 14% of the predator’s population (Tables 3, 4, Fig. 4H).

When respondents were asked about the snow leopard status in their area, 89% of the respondents were of the view that it is common while 9% thought that it is rare and 2% of the respondents claimed it to be absent in their area. Similarly, when respondents were asked about the status of grey wolf, 85% said that it common, 13% claimed it as a rare species while 2% claimed it absent in their area (Fig. 5G,H).

### Economic impact of predation on local population

Results indicates statistically significant values (p < 0.05) when contribution of livestock in household income was compared with depredation cases. The total economic loss due to depredation accounts for (US$ 67,311.75) between 2014 and 2019 (Table 1). Out of the total economic loss, the maximum loss was incurred from depredation of yaks that accounts for (US$ 32,000) followed by cattle (US$ 14,343.75) goats (US$ 10,750) and losses due to predation on sheep contributes, US$ 10,218.75. Most losses were due to predation from wolves, that account for US$ 34,625 followed by snow leopards with US$ 32,687.5 (Table 1).

### Perceptions towards carnivores

About 63% of respondents perceived snow leopard to be the most dangerous animal while 37% declared same for the grey wolf (Fig. 4B). Concerning reasons behind huge depredation losses in the valley, majority people (53%) attributed to loss of natural prey, some (24%) believed livestock is more exposed to the predators and easier to capture, while rest (23%) of the respondents claimed livestock to be the preferred prey (Fig. 4G). Despite ongoing human-predator conflicts in the area, about half (49%) respondents considered predators to be no important species of the area (Fig. 4A).

### Detection and seasonal patterns of predators

When asked about seasonality of the predation cases about 22% of respondents claimed that predation occurs in winter, 21% of the respondents claimed summer season as a threat, 17% said more predation cases occur in autumn season, 15% claimed spring season as a threat, 13% of the respondents are of the view that most of the predation cases occur in both spring and season and 12% of the respondents said that majority of the predation cases are recorded in summer, autumn and spring seasons (Fig. 5A).

### Challenges for conservation

About 69% of respondents perceived that there are low threats to snow leopard while 5% are of them opinion that the threats faced by the snow leopard are of medium nature and 26% of the respondents declared the threats faced by the snow leopard to be of severe nature. When asked about the nature of threats faced by wolf, 69% of the respondents are of the view that the threats faced by wolf are not severe while13% perceived the threats to be of medium nature and 30% respondents are of the view that the threats faced by the wolf are of severe nature (Fig. 4E,F).

In response to the effectiveness of grazing patterns about 49% of the respondents perceived predators proof corrals as the topmost conservation strategy while 32% considered guard dogs as a beneficial conservation strategy and 19% perceived guarded grazing as a conservation strategy (Tables 3,4, Fig. 5B). These protection measures are important because, majority shed (80%) in pasture are unprotected (Fig. 5C). Measure suggested by respondents to maintain wildlife population in their area included developing relevant rules and regulations (50% respondents), engagement of local community in conservation programs (37%), and enforcement existing wildlife laws, rules, and regulations (13%) (Fig. 5D). Majority herders (80%) did not any compensation for livestock losses, few (15%) which did, they received partial compensation (PKR 1000–6000). Only 5% respondents received substantial compensation (Fig. 5E,F).

## Discussion

### Patterns of livestock predation

In highly natural resources dependent areas around the globe, when a predator kills livestock, the herding communities retaliate^8,24^. Same is the case with Misgarities who rely mostly on livestock as one of the major livelihood options. The present study indicates that livestock numbers have increased between 2104 and 2019 with a record percent increase of 37.64% in the year 2019 (Table 2) possibly due decreasing number of livestock in other areas bordering Misgar like the Khunjerab Villagers, which are in agreement with the Parks and Wildlife Department of Gilgit-Baltistan that requires them to decrease the livestock number in order to qualify for conservation incentives in the form of tourist entry fee and income from sustainable trophy hunting programme. Thus, there is an increasing demand for meat from the adjacent communities and Misgarities are taking it as an opportunity. The current study also shows an increasing livestock depredation rates with 3.34% higher depredation rate as compared to other cases reported around the world^25,26^.

### Economic impact of predation on local population

The financial loss due to predation was considerably high, equating to 18% of an average household’s income in the study area. Similar amount of financial losses of livestock depredation by large carnivores have been reported from other locations in south Asia (e.g., 17% in China^26^, 19.8% in other areas of Pakistan^27^; 17% in Bhutan^18^; and 11% in India^28^). Income from livestock has a pivotal role in the livelihood of the respondents, who have minimal alternate earning sources. The damages caused by a predator to livestock thus creates a conflictual condition, that constitutes a major threat to wild carnivores in this area. However, this risk could be mitigated through sound conservation strategies that include compensation for the livestock losses.

### Perception towards carnivores

In the Karakoram-Pamir mountains, predation of livestock is one of the major issues with varying intensity. The spatial variations can be attributed to predators and prey abundance, herd size and herding practices and predator type. Snow leopard, wolf, and lynx are thought to be dangerous predators, while the brown bear is reported as less fatal to livestock in the region^8^. Similar perceptions were noticed during the current study. People regard snow leopard and wolf as dangerous animals, lynx was not recorded in the area, while brown bear was considered less fatal. Wild carnivores are known to prey selectively upon different livestock species to optimize their foraging strategy. The foraging behaviors accounts for the size of the predator and the size of their prey, prey preference, and abundance^29^. Prey preference observed during the current study could be explained under framework of optimal foraging. For example, we found that most of the sheep were preyed by wolf, while goats, cows and yaks were hunted by snow leopards. Being larger and stringer animal, snow leopards are probably going after larger preys.

### Seasonality in predation

Din et al*.*, reported average sighting of snow leopard (1.6 + 0.15) and wolf (3.9 ± 0.32) in the Pamir region, which overlaps with our study area. In concurrence with that, majority of respondents (82%) during the current study claimed 1–5 encounters with snow leopards, and remaining 18% had observed snow leopards more than 5 times. Similarly, 79% respondents sighted grey wolf 20 to 30 times, and 21% respondents sighted more than 30 times in their life (Fig. 4C,D). Higher encounter for wolves was possibly due to their wider distribution and group living behavior. Seasonality in predation is common phenomenon that widely documented for large carnivores^7,21,23^. For example, Li et al. reported predation in two seasons only; autumn (37.2%) and summer (36%). In Misgar, Autumn was a tie of lowest predation, while highest predations were recorded in winter, followed by spring and summer.

### Conservation implications

Human–wildlife conflict is one of the emerging challenge towards conservation of predators, specifically in underdeveloped countries^30^. Other interlinked threats include, persecution by herders, human population growth, reduction in wild prey-base and illegal hunting^31^. The present study documents that wildlife in Misgar is facing similar threats in greater intensity. Tested approaches towards mitigation of Human–wildlife conflict include improved animal husbandry, predator-proof corals, guarded grazing, habitat restoration, and promotion of co-existence with predators^21^. Community-based livestock insurance schemes along with awareness program can promote co-existence^32^. We believe that the aforementioned mitigation measures are appropriate for the Misgar valley, these should be initiated by the Government of Gilgit-Baltistan without further delay, in order to safeguard future of unique biodiversity of this pristine habitat. In current study, we have noted that the responded who were compensated for the losses have relatively positive attitude towards the predators and therefore they were not involved in retaliatory killings. Extreme climate events leading to disasters, soil erosion, drought, and ecosystem imbalance are also threatening the existence of wildife^33^ in the area, thus need attention of policy makers.

## Conclusion

The current study indicates that both the livelihoods of herder communities and population of the carnivores are at the stake due to Human–wildlife conflict and other associated threats in Misgar. Therefore, a robust Human–wildlife conflict management program at the state level, needs to be realized that addresses issues related to depredation losses through mitigation measures and provision of safety nets and promotes acceptance of predation through environmental education in the area.
